# Robotic surgery governance structures: a systematic review

**DOI:** 10.1007/s11701-025-02356-8

**Published:** 2025-05-15

**Authors:** Eoghan Burke, Michael Devine, Patricia Harkins, Sarah Fenn, Mohammad Faraz Khan, Barry B. McGuire

**Affiliations:** 1 Royal College of Surgeons in Ireland, Dublin, Ireland; 2 Royal College of Physicians in Ireland, Dublin, Ireland; 3 Mater Misericordiae University Hospital, Dublin, Ireland; 4 Department of Surgical Affairs, Professor of Surgical Education and Academic Development in The Royal College of Surgeons , Royal College of Surgeons in Ireland, St. Vincent’s University Hospital Dublin, Dublin, Ireland

**Keywords:** Robotic-assisted surgery, Governance structures, Training, Privileges

## Abstract

Since the Food and Drug Administration in the United States approved the first robotic platform for use in humans in 2000, there has been a steady increase in the popularity of robotic approaches to surgery. Robotic-assisted surgery (RAS) offers enhanced 3D visualisation of the operative field and increased flexibility and agility of the instruments, amongst other benefits. However, robotic platforms are complex systems and there is evidence that they may be associated with increased patient risk, particularly in the initial learning curve of a surgeon's robotic training. To ensure the continued safe use of RAS, adequate governance policies must be in place to regulate training, ensure patient safety and maximise the benefits of RAS programs. This systematic review synthesises all available evidence on RAS governance structures internationally for the first time. It was conducted and reported per the Preferred Reporting Items for Systematic Reviews and Meta-Analyses statement. This systematic review identified, for the first time, three guidelines offering recommendations on the Governance Structures for RAS programs. We extracted the key recommendations to provide a holistic set of guidelines. Through consensus, we offer recommendations for the composition of a RAS governance committee, the frequency with which the committee should sit, and the remit of the committee, including training, granting of privileges, quality assurance and continued professional development. We also stress future research needs in RAS team credentialing and vendor-agnostic training pathways. These recommendations may serve as a template for establishing future RAS programs.

**Supplementary Information:**

The online version contains supplementary material available at 10.1007/s11701-025-02356-8.

## Introduction

Since the Food and Drug Administration in the United States approved the first robotic platform for use in humans in 2000 [1], there has been a steady increase in the popularity of robotic approaches to surgery [2]. Indeed, across most disciplines, there has been a year-on-year increase in the number of surgeries performed using robotic platforms [3]. This trend is predicted to continue apace. Robotic-assisted surgery (RAS) offers enhanced 3D visualisation of the operative field and increased flexibility and agility of the instruments, amongst other benefits [4]. However, robotic platforms are complex systems and there is evidence that they may be associated with increased patient risk, particularly in the initial learning curve of a surgeon’s robotic training [5].

RAS differs significantly from conventional open or laparoscopic surgery regarding the operating room layout, the operating surgeon’s location, and the overall impact on team dynamics. The importance of adequate surgeon and team training is well documented; however, currently, this training is predominantly vendor-led with little input from international or national surgical governing bodies [6]. To ensure the continued safe use of RAS, adequate governance policies must be in place to regulate training, ensure patient safety and maximise the benefits of RAS programs. The governance structures in healthcare are defined as a formalised framework that outlines the roles, responsibilities, policies, and oversight mechanisms utilised in the delivery of a service [7]. Effective independent governance structures will allay potential ethical concerns of patients and healthcare professionals regarding vested interests. Currently, 12 robotic platforms are approved for use worldwide, and more are on the horizon. Thus, there is an increasing impetus to ensure that governance structures are robust and vendor-agnostic [8].

### Study aims

This systematic review aims to synthesise all available evidence on RAS governance structures internationally for the first time. It will focus on independent governance structures distinct from those established by industry.

## Materials and methods

This is a systematic review of Governance Guidelines for RAS programs. It was conducted and reported per the Preferred Reporting Items for Systematic Reviews and Meta-Analyses (PRISMA) statement [9]. Ethical approval was not required for this study. Registration with PROSPERO was sought; however, this form of review (on governance structures) is outside their remit for registration.

### Inclusion and exclusion criteria

All original research articles, abstracts, conference proceedings from the bibliographic database’s inception, or any grey literature from surgical societies and white papers were eligible for inclusion. Guidelines that did not explicitly discuss governance structures were excluded.

For this review, “governance structures” refer to documents outlining the roles, responsibilities, policies, and oversight mechanisms involved in delivering an RAS program in a hospital [7].

Orthopaedic surgery was excluded due to its unique integration with radiology and its distinct RAS platforms, which require specialised governance structures and collaboration with imaging specialists that fall outside the scope of this review.

While we acknowledge the vital role of industry in advancing robotic surgery technology, we excluded industry guidelines on RAS governance. Our decision was based on the belief that governance should remain independent of commercial interests to ensure clinical decisions prioritise patient safety over business motives.

### Search strategy

A detailed search strategy was developed and refined with a medical librarian. A preliminary search was conducted to identify key terms. The finalised search string developed included keywords and MeSH terms: governance OR governance processes OR governance committee OR governance guidelines AND robotic surgery OR robotic-assisted surgery. This search string was applied to the bibliographic databases PubMed, Web of Science, CINAHL, and EMBASE. No limitations were applied, and all databases were searched from inception until June 10th 2024. Grey literature was searched using the BASE database. Hand-searching references in the included guidelines was completed to identify any missed guidelines. A citation search using Google Scholar ensured all relevant studies were included. The search strategies for each database are provided in Appendix 1.

### Study selection

Following the removal of duplicates, all of the titles and abstracts were reviewed independently by two authors. The guidelines meeting the aforementioned inclusion criteria underwent full-text review. Conflicts about a guideline’s suitability were resolved by consensus. Following the full-text review, all suitable guidelines were brought forward for qualitative review.

A systematic review management system was used during this study [10].

### Data extraction

Two authors independently extracted the required data from the eligible studies using a predetermined data extraction form in Microsoft Excel. Any conflicts in the extracted data were resolved by consensus. The data extracted included the first author, year of publication, surgical society represented, recommendations about the composition of a RAS governance committee, recommendations on intervals for review meetings, and details on the suggested remit for a RAS governance committee, e.g. Education and training, granting privileges, audit of outcomes, and pathway for troubleshooting issues. Data about specific guidelines for training, granting of privileges and guidance on using proctors were also extracted.

## Results

### Study identification and selection

**Fig. 1:**
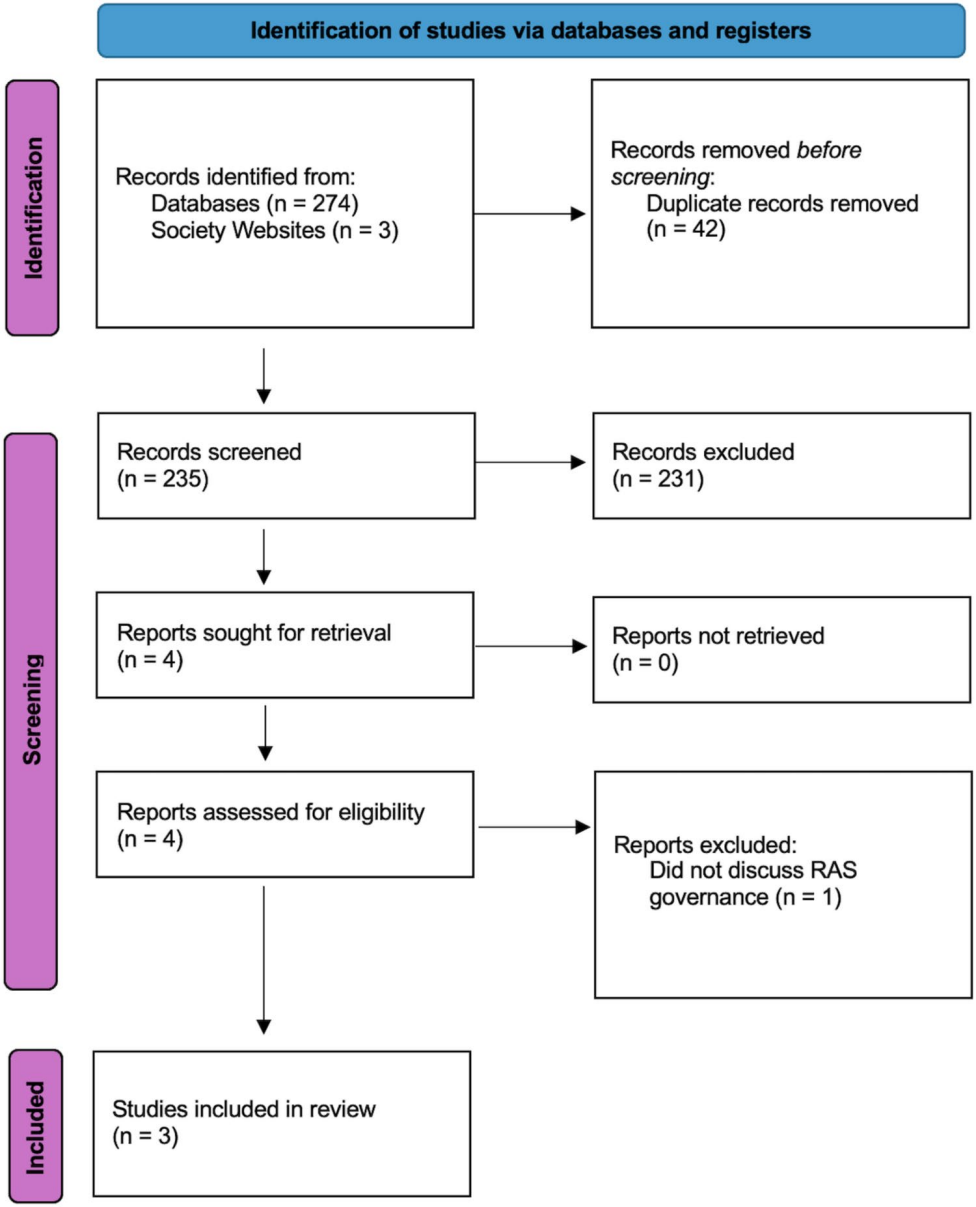
PRISMA flow diagram for study selection

After applying the search string to the aforementioned bibliographic databases, 274 papers were identified. These were uploaded to a systematic review manager. Forty-two duplicates were identified. Following the removal of these duplicates, 232 papers remained. These papers were screened independently by two of the authors. 231 were removed as they did not meet the inclusion criteria. Searching for grey literature identified three further papers. Following full-text review, three guidelines were identified as appropriate for inclusion in this qualitative analysis. This process is depicted in the PRISMA flow diagram (Fig. 1).

### Characteristics of included guidelines

An overview of the included guidelines is depicted in Table 1. Two guidelines were developed by national surgical societies, namely the Royal Colleges of Surgeons in Edinburgh [11] and England [12]; the remaining guideline consensus document was created by a speciality association [13], the Association of Upper Gastrointestinal Surgery of Great Britain and Ireland. The guidelines were all developed between 2022 and 2024.

**Table 1:** General characteristics of included studies and suggested remit of a robotic-assisted surgery governance group

Study ID	Year	Society	Composition of governance committee	Interval for meeting
Pucher et al. [13]	2024	The Association of Upper Gastrointestinal Surgery of Great Britain and Ireland	Senior robotic lead Specialty-specific lead	Not reported
Parks et al. [11]	2022	Royal College of Surgeons in Edinburgh, Robotic Surgery Taskforce	Robotic governance and peer review group (RGPG): Established robotic surgeons Anaesthetists Surgical care practitioners (SCPs) Operating theatre team Members (scrub teams, operating department practitioners [ODPs]) Representatives from the hospital management team A robotic surgery operational group (RSOG): Representatives from IT, medical physics, clinical engineering, sterile services, operating theatre stores and procurement	Not reported
Beard et al. [12]	2023	The Royal College of Surgeons of England–robotic surgery task force	Robotics Surgery Governance Group: Representatives from the surgical directorates undertaking robotic surgery, theatres, audit and governance lead	Three monthly basis
*Suggested remit of a robotic assisted surgery governance committee*				
Pucher et al. [13]	Prospective data collection Robotic programme-specific internal audit and review processes Ensure surgeons: complete an approved robotic curriculum and structured proctorship programme Monitor surgeon outcomes and competence			
Parks et al. [11]	Regulation of education and training Assessing requests for robotic surgical privileges Auditing of surgical outcome Auditing of cost effectiveness Approval of proctors Trouble shooting of issues with the RAS program			
Beard et al. [12]	Regulation of education and training of surgeon and entire theatre staff Assessing requests for robotic surgical privileges Auditing of surgical outcome Approval of proctors			

The guidelines recommended a dedicated RAS governance group within any hospital intending to perform RAS. Beard et al [12] were the only guideline to recommend the frequency with which the RAS governance group should meet: 3 monthly.

The three included guidelines gave recommendations about the remit of a RAS governance group. The overarching themes included:

- Regulation of surgeon training for RAS.
- Regulation of theatre team training for RAS.
- Granting of privileges to perform RAS.
- Approval of proctors.
- Audit of outcomes.
- Troubleshooting of problems arising in the RAS theatre.

The remit outlined by each guideline for a RAS governance group is summarised in Table 1.

### Recommendations on training, granting of privileges and proctorship

Table 2 displays the recommendations made by each guideline regarding RAS training programs, the granting of robotic privileges, the use of a structured proctorship model, and methods to ensure ongoing competence.

**Table 2:** Recommendations on training, granting of privileges, proctorship and ongoing competence assurance

Recommendation	Study		
	Pucher et al. [13]	Parks et al. [11]	Beard et al. [12]
Recommended robotic assisted surgery training program	Surgeon focussed Platform-specific manufacturer-approved curriculum Should include didactics, case observation, dry- and wet- lab training, followed by cadaveric or animal model operating, prior to proceeding to appropriately selected proctored cases	Surgeon and team focussed Technical and non-technical skills emphasis. Graduated training pathway: Theoretical self-directed online training modules Robotic surgical simulation practice Dedicated training on inanimate models, animal or cadaveric models Recommend attendance at a dedicated hands on course Practical hands on training for the surgical team–vendor led First assistant training–recommended for all surgeons, trainees and SCPs Procedure specific robotic surgical skills courses–specialty specific	Technical and non-technical skills. graduated training pathway: Self-directed online training, which should be a combination of generic and platform- agnostic skills alongside platform specific training Simulator based training: a minimum of nine hours and dexterity/accuracy scores above 90% for all parameters Observership (first assistant training) Co-operating via a dual console for a set number of procedures. essential that training sites have a dual console Sign off for further training based on platform proficiency to an oversight committee
Granting of privileges	Surgeons are expected to complete an approved robotic curriculum and structured proctorship programme	Not reported	Completion of graduated training program as outlined alongside sufficient proctorship: minimum requirement of 10 proctored cases unless robotic fellowship trained then 5 proctored cases
Proctorship	Proctors should be fully trained surgeons experienced in robotic surgery and accredited for the relevant robotic platform Proctoring process should continue until both proctor and proctee deem the surgery to be safe, independent, and competent Training should be competency-based, without set minimum or maximum case numbers	Proctor should be onsite at least for the initial cases Proctoring should continue until both the operating surgeon and proctor are satisfied that the procedure can be performed independently at a safe level	Every attempt should be made to utilise existing robotics-trained surgeons already employed in a department Mandatory requirements for robotics proctors: Already accredited to perform robotics surgery elsewhere and who had performed more than 100 robotics procedures (platform-specific) Personalised video library containing operative videos of procedures they intend to teach Should be on the vendors list of approved proctors Same proctor should be used for entirety of training Proctor must be of the same speciality and capable of taking over the surgery if needed Proctor must be comfortable in performing the same surgery laparoscopically +/− open. All proctors must have a letter of authority from human resources indicating indemnity cover Each proctored case would be signed off by the surgeon being trained, the proctor, the scrub nurse and the anaesthetist
Methods to ensure ongoing competence	Not reported	Surgeon will need to maintain an adequate volume of RAS cases annually–number of cases will be speciality dependent and guided by international practice Prospective audit of surgeon volume and outcomes	Evidence of competency should be based on agreed metrics, examples include: Completion of five core simulator skill exercises with a passing score of 90% every 2 years Case logs from recent two years must perform at least 20 procedures per year, in these 2 years Failure in the above will result in case- proctoring for the next two cases Surgeons who are inactive for more than 90 days must complete core simulator exercises with a passing score above 90% Surgeons performing more than 50 cases in two years will be exempt from the above

All three guidelines recommend a dedicated training pathway for surgeons pursuing RAS [11–13]. The guidelines agreed that a training program must be graduated from self-directed online learning modules to a structured proctorship program. However, there were discrepancies amongst the guidelines regarding whether the training should be surgeon-focussed or surgeon and team-focussed, whether it should consist of technical and non-technical skills training, and whether it should be vendor-led or vendor-agnostic (Table 2).

Concerning the granting of privileges, two of the guidelines provide recommendations. Pucher et al. [13] are broad in their guidance that surgeons should complete an approved robotic curriculum and structured proctorship program. Beard et al. [12] similarly advocate completing a graduated training program and make specific recommendations on the minimum number of proctored cases (Table 2).

The importance of proctorship in RAS training is highlighted in all three guidelines. Beard et al. [12], in particular, give specific guidelines for proctorship, including the use of a sufficiently experienced proctor (performance of over 100 platform-specific procedures), the use of the same proctor for the duration of a surgeon’s training and ensuring the proctor is of the same speciality and capable of taking over the surgery if needed (Table 2).

Parks et al. [11] and Beard et al. [12] stress the importance of ensuring ongoing, continuous professional development. Parks et al. state that prospective audit of a surgeon’s RAS volume and outcomes should be maintained and that all RAS surgeons should maintain a sufficient volume of cases annually. They do not specify indicative case numbers but state these should be speciality-specific and guided by international consensus. Beard et al. [12] provide detailed guidance about ongoing competency assessment. The recommendations include completing five core simulator skill exercises with a passing score of 90% every 2 years, surgeons must perform at least 20 procedures per year, and surgeons who are inactive for more than 90 days must complete core simulator exercises with a passing score above 90%. They stipulate that any surgeon performing 50 cases in the prior 2 years would be exempt from these criteria (Table 2).

## Discussion

### Recommendations from current international guidelines

Following this systematic review, the following key recommendations were extracted from the guidelines. The authors agreed upon these recommendations by consensus. They may serve as a guideline for establishing RAS Governance Committees both nationally and internationally.

### Recommendations for the composition of a RAS governance committee, their remit and interval for meeting

All three of the included guidelines recommended the formation of a dedicated RAS governance group in any hospital undertaking or intending to undertake RAS [11–13] and made recommendations regarding the composition of such a group.

**Fig. 2:**
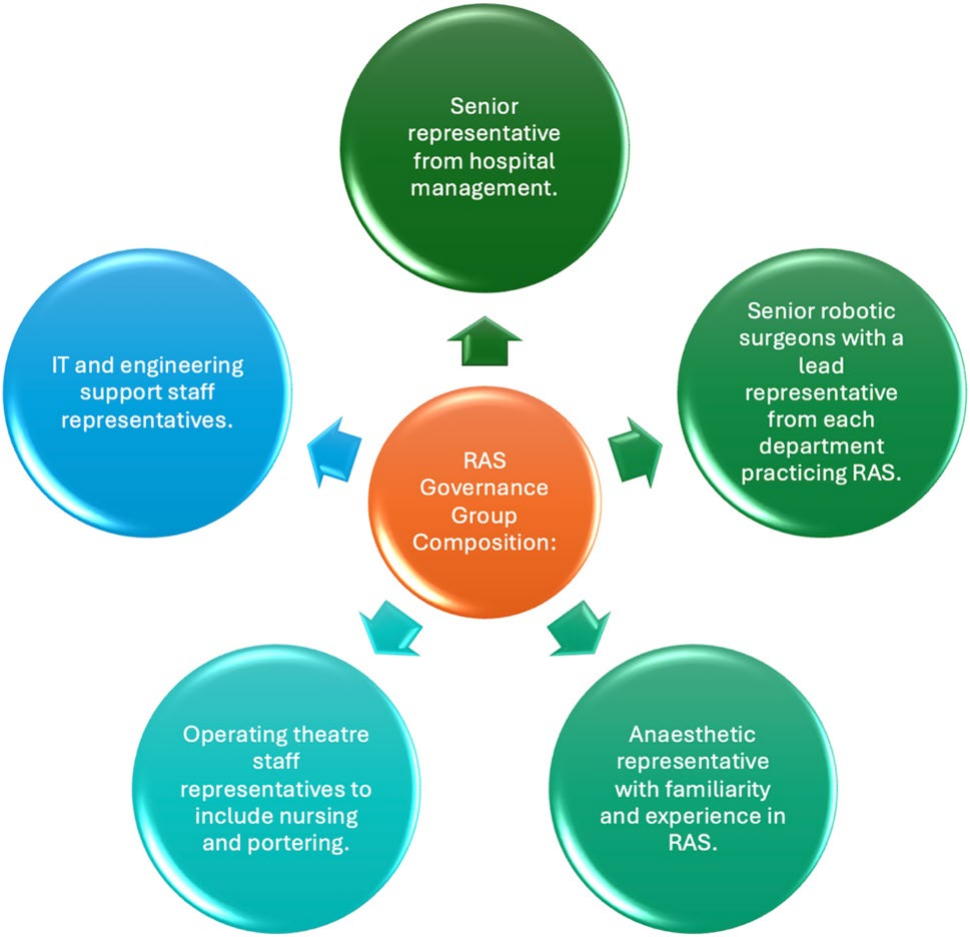
Recommendations for the composition of a RAS governance group

Incorporating the findings of these three documents, we propose the mandatory inclusion of the following stakeholders into RAS governance committees (Fig. 2). These include:

- A senior executive from hospital management.
- Senior robotic surgeons with a lead representative from each department practising RAS.
- An anaesthetic representative with experience in RAS.
- Operating theatre staff, including RAS CNM, Portering, and CSSD staff.
- IT and engineering support staff representatives.

**Fig. 3:**
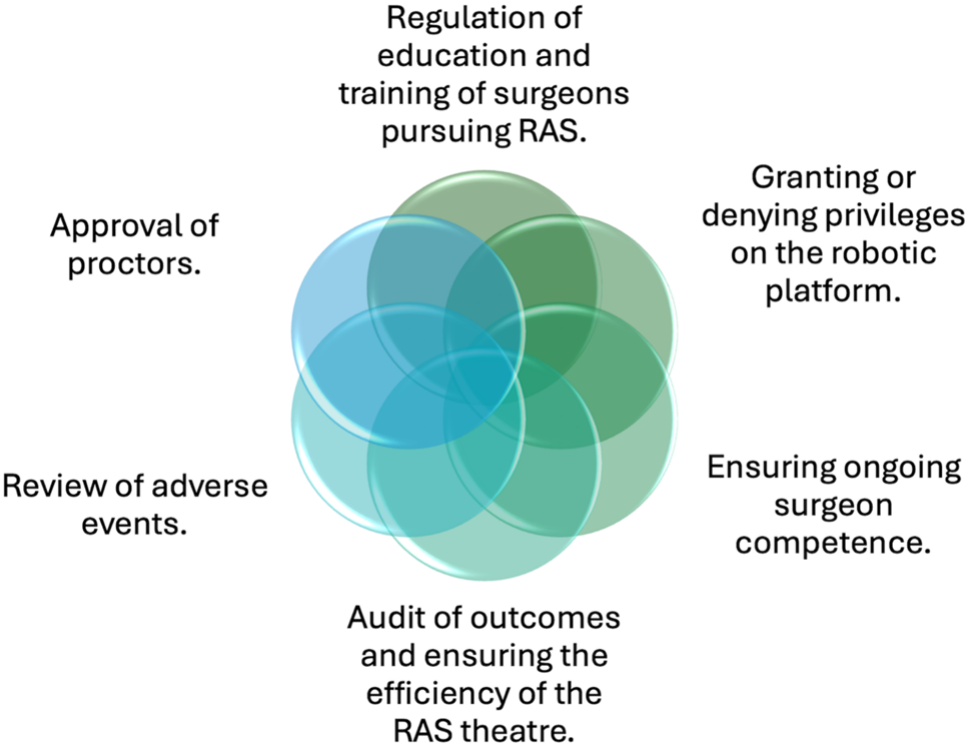
Suggested remit of a RAS governance group

The remit for a RAS governance group should contain the following core domains as broadly described by all three guidelines [11–13] (Fig. 3):

- Regulation of education and training of surgeons pursuing RAS.
- Granting of privileges to perform RAS.
- Approval of proctors.
- Ensuring ongoing surgeon competence.
- Audit outcomes and ensure the efficiency of the RAS theatre.
- Review of adverse events.

Regarding the interval for RAS governance group meetings, 3 monthly basis should be considered a minimum and revised depending on individual centres’ needs. In the initial period of establishing a RAS program, it is conceivable that meetings will need to be held more often to deal with issues as they arise. There should also be a mechanism to trigger a meeting in the event of adverse events or operational issues that need to be urgently addressed. The criteria and mechanism for triggering such meetings should be established as a priority by the RAS governance group.

### Recommendations on training, granting of privileges and proctorship

There is an increasing focus on developing curricula to train future surgeons in RAS [14]. Ideally, these curricula will be platform-agnostic and endorsed by the relevant surgical societies [15]. An example of such a curriculum is the Fundamentals of Robotic Surgery skills curriculum (FRS), developed using a Full Life Cycle Curriculum Development process during four consensus conferences involving 66 subject matter experts [16]. The FRS curriculum focuses on seven unique robotic tasks that address 25 technical skills necessary to perform robotic surgery proficiently and safely. The curriculum also encompasses non-technical training aspects of team communication and teamwork in RAS. The FRS curriculum is now standard in select simulators such as the RobotiX Mentor by Surgical Science [17]. Thus, the curriculum could be deployed in a region to standardise RAS training.

**Fig. 4:**
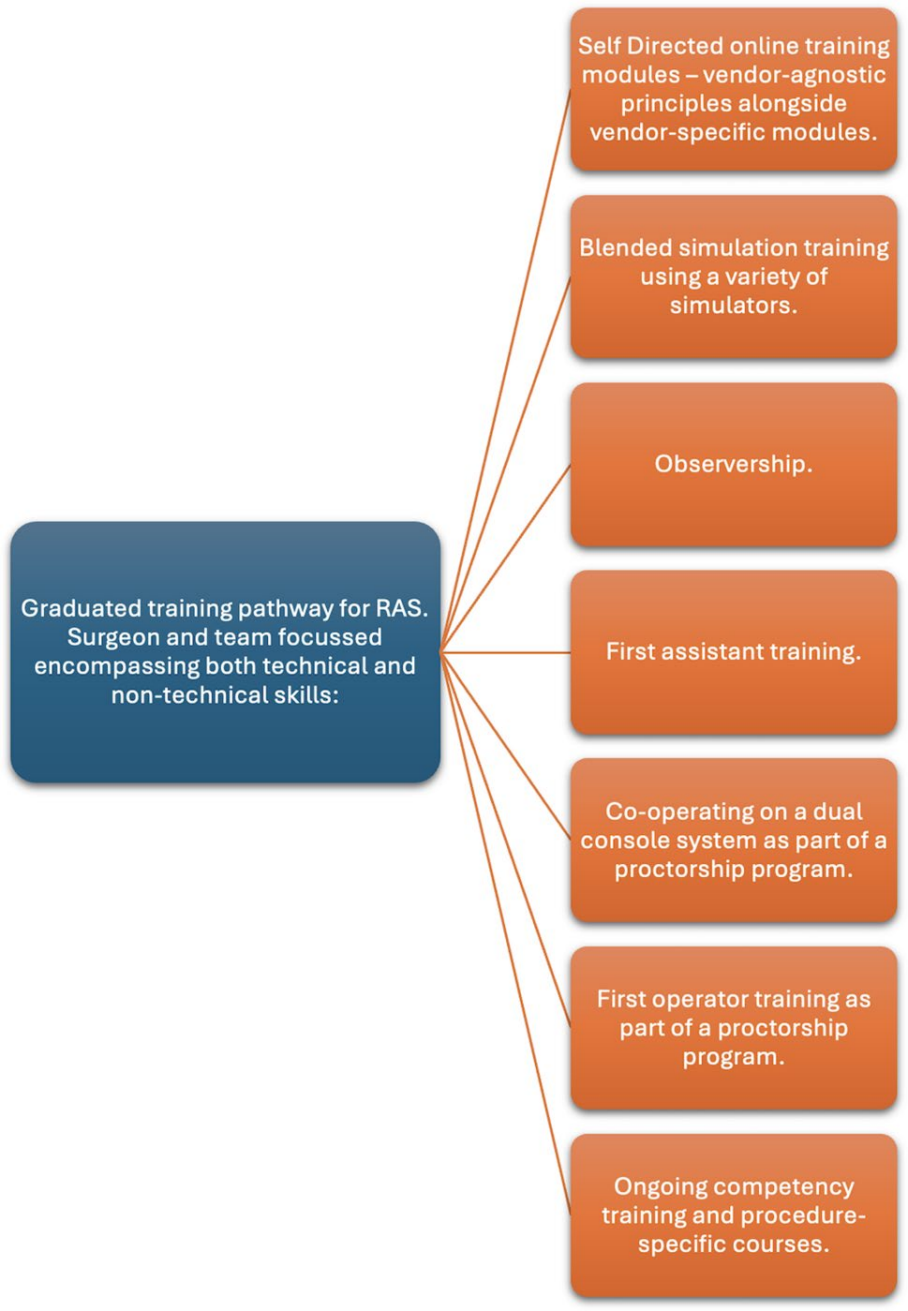
Graduated training program towards independent RAS. Focus on surgeon and team training with both technical and non-technical skills acquisition

This is an area of ongoing research and debate. We recommend the following broad approach to a structured training program, depicted in Fig. 4, which will be subject to and guided by ongoing research.

As discussed above, a vital remit of the RAS governance group will be assessing applications for privileges to perform RAS. Without an internationally recognised and approved training pathway for RAS, we recommend the following guidance regarding granting privileges: the candidate should have documentary evidence of completion of a graduated training program in RAS. The training program should cover RAS’s technical and non-technical aspects and display the progression of competence from didactic learning to console operating as the primary surgeon. The applicant should also display evidence of a structured proctorship program. The proctor, the proctee, the anaesthetist and the theatre CNM should sign off on each proctored case. The proctorship should continue until there is consensus that the proctee is competent and safe to operate independently.

The RAS governance committee should be instrumental in approving a suitable proctor. Proctors should be deemed experts in the given discipline and the generality of robotic surgery [18]. A crude cut-off of performing at least 50 procedures using the robotic platform is often used as a benchmark however, as the use of RAS continues to expand, this criterion is likely to change, as was seen with the adoption of laparoscopic surgery. The proctors should also be proficient in performing the given surgery laparoscopically and open if an emergency necessitates a conversion. The same proctor should ideally be used throughout a surgeon’s training, ensuring proctor-proctee familiarity and efficient training progression. Currently, the vendor recommends proctors, which raises the possibility of conflicts of interest and bias towards competency evaluation on a given platform. As robotic surgical experience grows within an institution or a region, proctors from within a department may be utilised, which may reduce the risk of conflicts of interest in using vendor-endorsed proctors.

Our study is the first systematic review of governance structures for RAS programs, but several limitations must be considered. The limited number of studies identified reflects the emerging nature of this field rather than a flaw in our search strategy. To ensure comprehensive coverage, we adhered to best practices for systematic reviews, including searching grey literature in the BASE database and conducting citation searches via Google Scholar. While the small number of papers is a limitation, we believe it highlights the lack of research in this area and encourages future exploration.

Additionally, we excluded industry-produced guidelines, which may be seen as a limitation due to the essential role of industry collaboration in introducing RAS programs. However, we prioritised ethical considerations, opting for unbiased, vendor-agnostic recommendations. We believe industry should not influence the governance of RAS programs within hospitals, though ongoing collaboration between hospitals and vendors is encouraged. In Fig. 2, we suggest that representatives from the hospital’s IT and engineering departments be part of the RAS governance group, liaising with industry on technical needs while operating theatre staff handle in-service training coordination with industry representatives.

This review focuses on governance for general and urological RAS programs. The unique needs and platforms used in orthopaedic RAS warrant separate review and a distinct analysis beyond the scope of this work.

## Conclusion

RAS is being adopted at an ever-increasing pace. It involves unique theatre dynamics and poses unique patient safety issues. The safe and efficient adoption of this technology requires rigorous governance structures. We present the first systematic review of current governance structures and make recommendations to guide future RAS programs. Ongoing research is needed to develop vendor-agnostic training curricula and methods to assess RAS team credentialing.

## Supplementary Information

Below is the link to the electronic supplementary material.

Supplementary file1 (DOCX 14 kb)

## Data Availability

No datasets were generated or analysed during the current study.

## References

[1] Bodner J, Augustin F, Wykypiel H, Fish J, Muehlmann G, Wetscher G et al (2005) The da Vinci robotic system for general surgical applications: a critical interim appraisal. Swiss Med Wkly 135(4546):674 16453207 10.4414/smw.2005.11022

[2] Harji D, Houston F, Burke J, Griffiths B, Tilney H, Miskovic D et al (2023) The current status of robotic colorectal surgery training programmes. J Robot Surg 17(2):251–63 35657506 10.1007/s11701-022-01421-w

[3] Halabi WJ, Kang CY, Jafari MD, Nguyen VQ, Carmichael JC, Mills S et al (2013) Robotic-assisted colorectal surgery in the United States: a nationwide analysis of trends and outcomes. W J Surg 37:2782–90 10.1007/s00268-013-2024-7 23564216

[4] Reddy K, Gharde P, Tayade H, Patil M, Reddy LS, Surya D (2023) Advancements in robotic surgery: a comprehensive overview of current utilizations and upcoming frontiers. Cureus 15(12) 10.7759/cureus.50415 PMC10784205 38222213

[5] Parsons JK, Messer K, Palazzi K, Stroup SP, Chang D (2014) Diffusion of surgical innovations, patient safety, and minimally invasive radical prostatectomy. JAMA Surg 149(8):845–51 24990549 10.1001/jamasurg.2014.31 PMC4373702

[6] Weber J, Catchpole K, Becker AJ, Schlenker B, Weigl M (2018) Effects of flow disruptions on mental workload and surgical performance in robotic-assisted surgery. W J Surg 42:3599–607 10.1007/s00268-018-4689-4 29845381

[7] Jalilvand MA, Raeisi AR, Shaarbafchizadeh N (2024) Hospital governance accountability structure: a scoping review. BMC Health Serv Res 24(1):47 38200541 10.1186/s12913-023-10135-0 PMC10777527

[8] Marchegiani F, Siragusa L, Zadoroznyj A, Laterza V, Mangana O, Schena CA et al (2023) New robotic platforms in general surgery: what’s the current clinical scenario? Medicina 59(7):1264 37512075 10.3390/medicina59071264 PMC10386395

[9] Moher D, Shamseer L, Clarke M, Ghersi D, Liberati A, Petticrew M et al (2015) Preferred reporting items for systematic review and meta-analysis protocols (PRISMA-P) 2015 statement. Syst Rev 4:1–9 25554246 10.1186/2046-4053-4-1 PMC4320440

[10] Ouzzani M, Hammady H, Fedorowicz Z, Elmagarmid A (2016) Rayyan-a web and mobile app for systematic reviews. Syst Rev 5:1–10 27919275 10.1186/s13643-016-0384-4 PMC5139140

[11] Parks W, R. Development of new robotic surgical services A guide to good practice 2022 https://fpc.rcsed.ac.uk/fpc-news/2022/may/development-of-new-robotic-surgical-services

[12] al. DBe. Robotic-assisted surgery: a pathway to the future 2023 https://www.rcseng.ac.uk/standards-and-research/standards-and-guidance/good-practice-guides/robotic-assisted-surgery/

[13] Pucher P, Maynard N, Body S, Bowling K, Chaudry MA, Forshaw M et al (2024) Association of upper GI surgery of Great Britain and Ireland (AUGIS) Delphi consensus recommendations on the adoption of robotic upper GI surgery. Ann R Coll Surg Engl 8:688–693 10.1308/rcsann.2024.0014 PMC11528368 38445587

[14] Chen R, Rodrigues Armijo P, Krause C, Force SRT, Siu K-C, Oleynikov D (2020) A comprehensive review of robotic surgery curriculum and training for residents, fellows, and postgraduate surgical education. Surg Endosc 34:361–7 30953199 10.1007/s00464-019-06775-1

[15] Khan MTA, Patnaik R, Lee CS, Willson CM, Demario VK, Krell RW et al (2023) Systematic review of academic robotic surgery curricula. J Robot Surg 17(3):719–43 36413255 10.1007/s11701-022-01500-y

[16] Satava RM, Stefanidis D, Levy JS, Smith R, Martin JR, Monfared S et al (2020) Proving the effectiveness of the fundamentals of robotic surgery (FRS) skills curriculum: a single-blinded, multispecialty, multi-institutional randomized control trial. Ann Surg 272(2):384–92 32675553 10.1097/SLA.0000000000003220

[17] Whittaker G, Aydin A, Raison N, Kum F, Challacombe B, Khan MS et al (2016) Validation of the RobotiX mentor robotic surgery simulator. J Endourol 30(3):338–46 26576836 10.1089/end.2015.0620

[18] Santok GD, Raheem AA, Kim LH, Chang K, Chung BH, Choi YD et al (2016) Proctorship and mentoring: its backbone and application in robotic surgery. Investig Clin Urol 57(Suppl 2):S114–S20 27995215 10.4111/icu.2016.57.S2.S114 PMC5161014

