# Supplementary material for: Robotic surgery governance structures: a systematic review
Source: J Robot Surg. 2025 May 15;19(1):218. doi: 10.1007/s11701-025-02356-8 (PMC12081519; doi:10.1007/s11701-025-02356-8)
Supplement: Supplementary file 1 — Supplementary file1 (DOCX 14 kb) [file 11701_2025_2356_MOESM1_ESM.docx]

***Appendix 1:***

Search Strategies for each bibliographic database: All searched from inception to a final search date 10^th^ June 2024.

***Pubmed:***

("governance"[Title/Abstract] OR "governance processes"[Title/Abstract] OR "governance committee"[Title/Abstract] OR "governance guidelines"[Title/Abstract])

AND

("robotic surgery"[Title/Abstract] OR "robotic-assisted surgery"[Title/Abstract] OR "Robotic Surgical Procedures"[MeSH])

***EMBASE:***

(governance:ti,ab OR "governance processes":ti,ab OR "governance committee":ti,ab OR "governance guidelines":ti,ab)

AND

('robot assisted surgery'/exp OR "robotic surgery":ti,ab OR "robotic-assisted surgery":ti,ab)

***CINAHL:***

(governance OR "governance processes" OR "governance committee" OR "governance guidelines")

AND

("robotic surgery" OR "robotic-assisted surgery")

***Web of Science:***

TS=(governance OR "governance processes" OR "governance committee" OR "governance guidelines")

AND

TS=("robotic surgery" OR "robotic-assisted surgery")

***BASE database of Grey literature:***

("governance" OR "governance processes" OR "governance committee" OR "governance guidelines")

AND

("robotic surgery" OR "robotic-assisted surgery")
